# The cytokines within the carotid plaque in symptomatic patients with internal carotid artery stenosis

**DOI:** 10.1186/1749-8090-9-139

**Published:** 2014-08-15

**Authors:** Dariusz Janczak, Piotr Ziolkowski, Jerzy Garcarek, Dawid Janczak, Karolina Dorobisz, Mariusz Chabowski

**Affiliations:** Department of Surgery, 4th Military Academic Hospital, 5 Weigla street, 50-981 Wroclaw, Poland; Department of Patomorphology, Wroclaw Medical University, 1 Marcinkowski street, 50-368 Wroclaw, Poland; Department of Interventional Radiology, Wroclaw Medical University, 213 Borowska street, 50-556 Wroclaw, Poland; Department of Clinical Proceedings, Faculty of Health Science, Wroclaw Medical University, 5 Bartla street, 51-618 Wroclaw, Poland

**Keywords:** Cytokines, Inflammatory reaction, Carotid plaque instability, Carotid artery stenosis, Carotid endarterectomy (CEA)

## Abstract

**Materials and methods:**

The experiment was carried out on 100 symptomatic patients with internal carotid artery stenosis that underwent carotid endarterectomy. Every patient had the wall of the carotid artery resected during organ harvesting surgery in order to evaluate some cytokines (TGF-β, VEGF, FGF, TNF-α) and to perform the immunohistochemistry (IHC). An immunoreactive score (IRS) was calculated based on the staining intensity and the number of cells stained. Over a 3-year period, 7 patients died, and 2 patients were lost to follow-up. The study group consisted of 91 patients. The control group comprised 20 young organ donors with confirmed death brain, who had their normal carotid artery sampled.

**Results:**

In all healthy donors (control group) with normal carotid arteries the three cytokines (TGF-β, VEGF, TNF-α) were not discovered. The presence of FGF was confirmed in 25% of healthy donors, probably due to an intima fibroblasts activity, responsible for the synthesis of elastin and collagen to the extracellular matrix (ECM). Only three cytokines (TGF-β, FGF, TNF-α) were found within atheromatous plaques (study group).

**Conclusions:**

Our research confirmed that these factors may accelerate the development of atheromatic plaque and its destabilisation.

The aim of the study was the evaluation of the inflammatory cytokines within atheromatic carotid plaque.

**Electronic supplementary material:**

The online version of this article (doi:10.1186/1749-8090-9-139) contains supplementary material, which is available to authorized users.

## Background

Cytokines are protein and peptide signaling molecules used in intercellular communication[1–5]. Cytokines are characterised both by renundancy (i.e. many cytokines have similar functions) as well as by pleiotropism[6, 7]. They may increase the smooth muscle proliferation by myocytes as well as synthesis and release of collagen and elastin to extracellular space by fibroblasts[8]. Such processes may cause the human atheromatous plaque enlargement and destabilization, which may lead directly to stroke[9]. Despite many experimental and clinical trials it is still an open question which cytokines are responsible for the development and destabilization of the carotid plaque[10].

### The aim of the study

The comparison of some cytokines concentrations within atheromatic carotid plaque and normal carotid artery.

## Methods

Having obtained the approval of the bioethics committee of the Wroclaw Medical University (No 739/2003), the experiment was carried out on 100 consecutive symptomatic patients (71 men and 29 women), aged between 46 and 79 years (mean age was 65.5), with critical internal carotid artery (ICA) stenosis. The authors received written informed patient consents to perform this study. All the patients had their history taken, had routine biochemistry lab tests performed, and the Doppler sonography with the ateromatous plaque, the flow parameters and the stage of carotid stenosis recorded. All the patients had brain CT or MRI examination. The patients were qualified to surgery according to NASCET and ECBT standards, i.e. those having substantial hemodynamic internal carotid artery stenosis of 70% or more. They underwent carotid endarterectomy (CEA) with patch angioplasty or eversion CEA. All the patients had one or more TIA episodes, 36% of them had at least one stroke episode, and 64% did not have any symptoms of a stroke. The patients who were diagnosed with diabetes or with cancer were excluded from the study. During the endarterectomy each patient had the part of internal carotid artery wall excised to examine the presence of some cytokines: transforming growth factor (TGF-ß), vascular endothelial growth factor (VEGF), fibroblast growth factor (FGF), and tumor necrosis factor (TNF-α). The immunohistochemistry (IHC) was performed as well. Over a 3-year period, 7 patients died, and 2 patients were lost to follow-up. The study group consisted of 91 patients. All the data were statistically analyzed by means of the Pearson chi-squared (*χ* 2) test, and non-parametric Mann–Whitney *U* test.

The control group consisted of 20 young organ donors (12 men and 8 women), aged between 20 and 28 years (mean age was 25) with confirmed death brain by the brain death committee. During organs harvesting surgery they had their normal internal carotid artery sampled as well.

The presence of the cytokines was assessed by semi-quantitative immunohistochemical method (antibody/cytokine) in four step scale: 0, 1+, 2+, 3+ via analysis of the grade of staining intensity. The immunoreactive score (IRS) was applied, as described by Remmele and Stegner (1987). The IRS is the effect of staining intensity: 0 – no reaction (no positive cells), 1–2 weak reaction (<10% positive cells), 3–4 intermediate reaction (10-50% positive cells), 6–12 strong reaction (>50% positive cells).

## Results

In all healthy donors of the control group the three cytokines (TGF-β, VEGF, TNF-α) were not discovered. In 25% healthy donors (5 people) the presence of FGF was confirmed by weak positive IHC reaction. In 75% healthy donors (15 people) the presence of FGF was not found.

**Figure 1:**
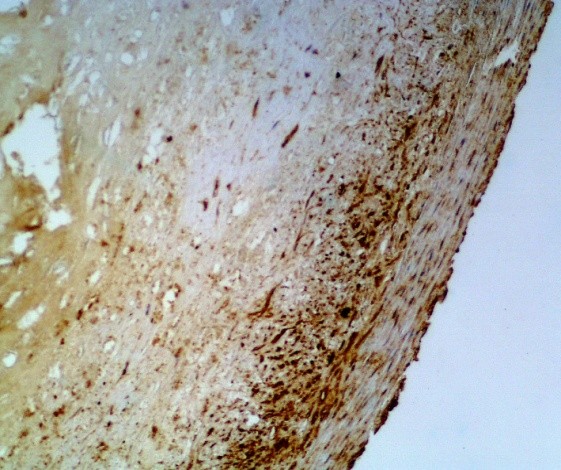
**The IHC of the ICA.** The presence of the cytokine TGF-ß (3+, strong reaction) according to the Remmele scale (IRS 12) (magnification ×200).

**Figure 2:**
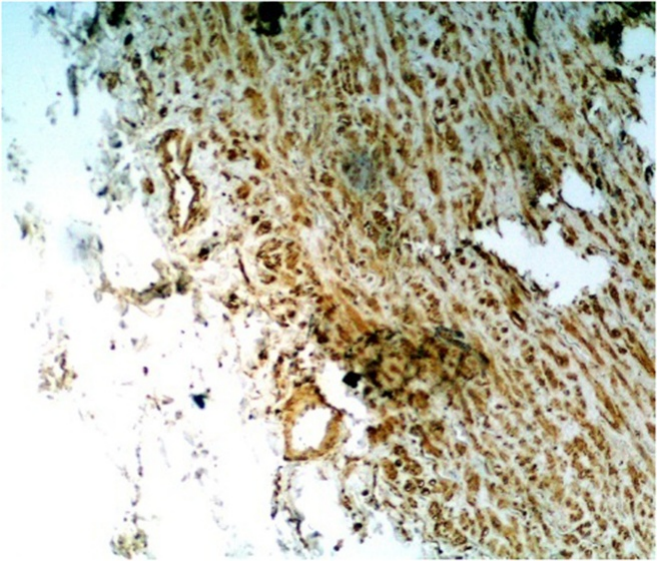
**The IHC of the ICA.** The presence of the cytokine VEGF (+, weak reaction) according to the Remmele scale (IRS 2) (magnification ×200).

**Figure 3:**
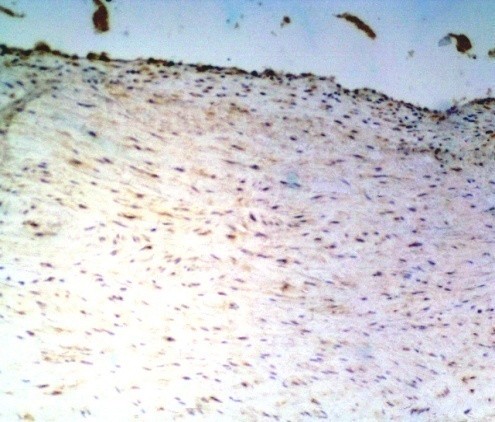
**The IHC of the ICA.** No cytokine FGF (0, no reaction) according to the Remmele scale (IRS 0) (magnification ×200).

**Figure 4:**
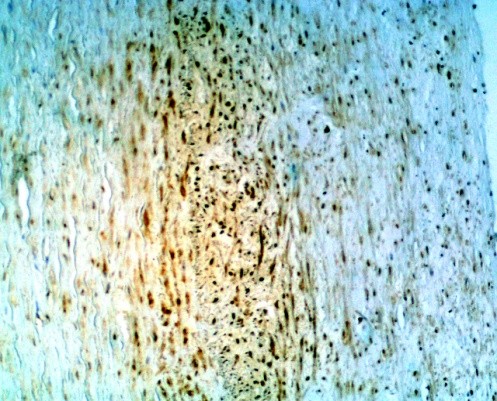
**The IHC of the ICA.** The presence of the TNF-α (2+, intermediate reaction) according to the Remmele scale (IRS 6) (magnification ×200).

In the study group no cytokines was discovered in 10 (11%) patients, and all four cytokines were present in 6 (7%) patients.As regards TGF-β cytokine: no cytokine was found in 57 (63%) patients, weak reaction was present in 30 pts, intermediate reaction – in 2 pts, and strong reaction – in 2 pts (Figure 1). In total TGF-β cytokine was present in 37% patients.As regards VEGF cytokine: no cytokine was found in 70 (77%) patients, weak reaction was present in 15 pts (Figure 2), intermediate reaction – in 6 pts, and no strong reaction was revealed. In total VEGF cytokine was present in 23% patients.As regards FGF cytokine: no cytokine was found in 12 (13%) patients (Figure 3), weak reaction was present in 30 pts, intermediate reaction – in 27 pts, and strong reaction – in 22 pts. In total FGF cytokine was present in 79 (87%) patients. In 49 (54%) pts there was increased FGF (intermediate or strong) reaction revealed. It should be remembered that cytokine FGF was present in 25% donors of the control group as well.As regards TNF-α cytokine: no cytokine was found in 55 (60%) patients (Figure 4), weak reaction was present in 27 pts, intermediate reaction – in 7 pts (Figure 4), and strong reaction – in 2 pts. In total TNF-α cytokine was present in 36 (40%) patients.

FGF cytokine was the most frequent cytokine within the atheromatous ICA wall. On the contrary, the rarest cytokine was the VEGF. The level of FGF cytokine was three times as high as the others (TNF-α, TGF-β, and VEGF).

The presence of some cytokines (TNF-α, TGF-β, FGF, VEGF) within atheromatous ICA was proved. The cytokine VEGF accelerates endothelial regeneration. TNF-α is a cytokine that contributes to a necrosis and proteolysis processes. The increased level of FGF is due to endothelial and intimal myofibroblast (myoFb) activity of the atheromatous ICA. It is responsible for the synthesis of elastic fibers and collagen, then accumulated in the atheromatous plaque, leading directly to carotid stenosis and destabilization of the plaque. The presence of VEGF was noted in patients with ICA stenosis without ulceration or destabilization of the plaque. On the contrary, FGF was found in patients with critical ICA stenosis due to plaque ulceration. TNF-α was noted in patients with plaque ulcerations, with endothelium and intima proteolysis, which leads to destabilization of the plaque. In all the patients the level of FGF correlated with the stage of the ICA stenosis. TGF-β showed the strongest and statistically significant correlation with the level of FGF (R = 0.372). FGF is significantly correlated with both atheromatous lesions proved in H&E stain (R = 0.360), as well as advanced plaque (R = 0.301). A statistically significant correlation was found between the clinical presentation of TIA, and cytokines (FGF, TGF-β). TNF-α co-existed with FGF, so it similarly correlated with atheromatous lesions in H&E stain, the stage of the ICA stenosis, and TIA symptoms.

What is the relationship between these cytokines and clinical presentations?

TGF and TNF-α have been reported to increase after stroke. A statistically significant correlation between stroke and the presence of TGF-β (R = 0.453), TNF-α (R = 0.624), and FGF (R = 0.476) was recorded. A statistically significant correlation between TIA and the presence of TGF-β (R = 0.403), TNF-α (R = 0.733), and FGF (R = 0.618) was also demonstrated. Results reported herein show no correlation between symptoms and the levels of VEGF.

A loose connective tissue, of high cellularity, with spindle-shaped cells, resembling myocytes (myofibroblasts and fibroblasts) was found in the atheromatous plaques with increased level of TGF-β, FGF, TNF-α cytokines, not VEGF. What is more, the large focuses of necrosis, calcium and cholesterol deposits, ulceration and disruption of the fibrous cap were observed. Fibrinogen was present within all arterial layers, and the smooth muscle cells were more frequently found.

## Discussion

No cytokines in the ICA wall of the control group may be a sign of not damaged intima, with no need for regeneration (VEGF), and no necrosis (TNF-α). The presence of FGF in some healthy donors was probably due to an intima fibroblasts activity, responsible for the synthesis of elastic fibers and collagen to the extracellular matrix (ECM)[11–13]. The continuous arterial blood pressure interacting on the vessel wall causes the need of an intima elastic fibers and collagen regeneration[14–16]. Results of our study underline the basic role of the FGF in the formation of the primary carotid plaque. FGF plays an important role in the remodeling of the extracellular matrix, and therefore may influence the clinical presentation[17–19]. The statistically significant impact of TGF-β on FGF has been observed as well. FGF and TNF-α influence the TIA and stroke occurrence, so these cytokines must participate in destabilisation of carotid plaque.

However, the definitive role of cytokines and the extracellular matrix (ECM) in pathogenesis of peripheral arterial occlusive disease is still unknown[20–23]. Therefore, there is a need for a further experimental and clinical studies.

## Conclusion

The presence of some cytokins (FGF, TGF-β, and TNF-α) within atheromatic plaque of the internal carotid artery stenosis may accelerate the development of atheromatic plaque and its destabilisation.

## Authors’ original submitted files for images

Below are the links to the authors’ original submitted files for images. Authors’ original file for figure 1 Authors’ original file for figure 2 Authors’ original file for figure 3 Authors’ original file for figure 4

## Abbreviations

CEA: Carotid endarterectomy
TIA: Transient ischemic attack
IRS: Immunoreactive score
IHC: Immunohistochemistry
ICA: Internal carotid artery
TNF-α: Tumor necrosis factor
TGF-β: Transforming growth factor
ECM: Extracellular matrix
VEGF: Vascular endothelial growth factor.
