# Supplementary figures and images for: The cytokines within the carotid plaque in symptomatic patients with internal carotid artery stenosis
Source: J Cardiothorac Surg. 2014 Aug 15;9:9060. doi: 10.1186/1749-8090-9-139 (PMC4283111; doi:10.1186/1749-8090-9-139)

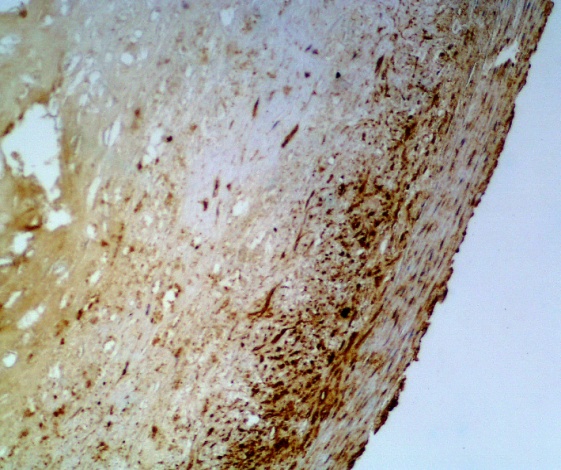

Supplement: Supplementary file 1 — Authors’ original file for figure 1 [file 13019_2014_1500_MOESM1_ESM.jpeg]

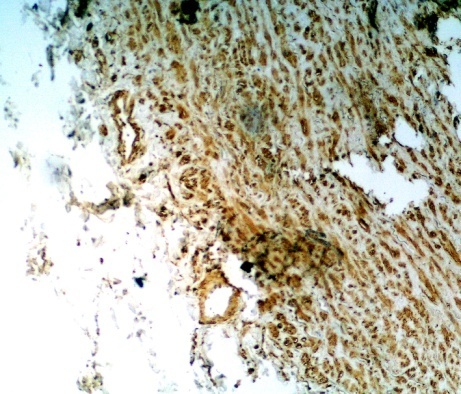

Supplement: Supplementary file 2 — Authors’ original file for figure 2 [file 13019_2014_1500_MOESM2_ESM.jpeg]

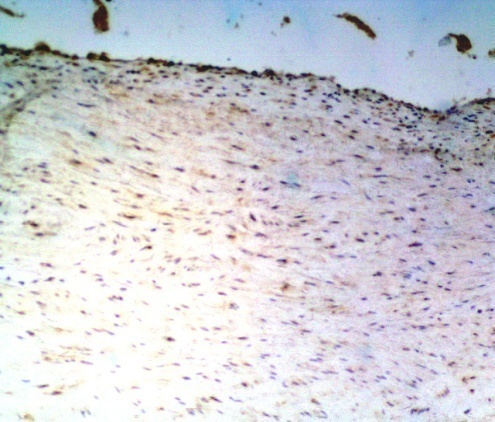

Supplement: Supplementary file 3 — Authors’ original file for figure 3 [file 13019_2014_1500_MOESM3_ESM.jpeg]

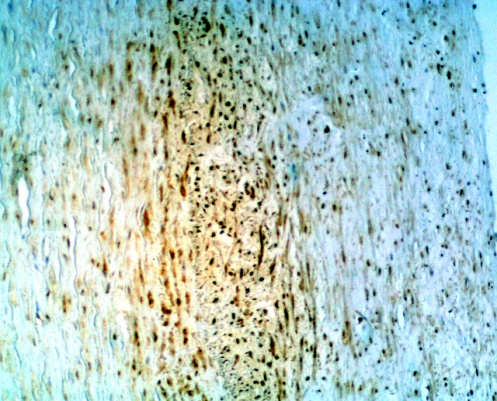

Supplement: Supplementary file 4 — Authors’ original file for figure 4 [file 13019_2014_1500_MOESM4_ESM.jpeg]
